# The Associated With Carbon Conversion Rate and Source–Sink Enzyme Activity in Tomato Fruit Subjected to Water Stress and Potassium Application

**DOI:** 10.3389/fpls.2021.681145

**Published:** 2021-06-16

**Authors:** Anrong Luo, Chenni Zhou, Jinliang Chen

**Affiliations:** ^1^Center for Agricultural Water Research in China, China Agricultural University, Beijing, China; ^2^Institute of Tibet Plateau Ecology, Tibet Agriculture and Animal Husbandry University, Nyingchi, China; ^3^Key Laboratory of Forest Ecology in Tibet Plateau (Tibet Agriculture and Animal Husbandry University), Ministry of Education, Nyingchi, China

**Keywords:** sucrose-metabolizing enzymes, starch metabolism enzymes, carbon conversion rate, tomato, potassium, water stress

## Abstract

Carbon metabolism in higher plants is a basic physiological metabolism, and carbon allocation and conversion require the activity of various enzymes in metabolic processes that alter the content and overall composition of sugars in the sink organ. However, it is not known how various enzymes affect carbon metabolism when tomato plants are subjected to water stress or treated with potassium. Although the process of carbon metabolism is very complex, we used the carbon conversion rate to compare and analyze the enzyme activities related to sugar metabolism and find out which carbon conversion rate are the most important. Results showed that water stress and potassium increased carbon import flux in the fruit, which was beneficial to carbon accumulation. Water deficit increased the activity of sucrose synthase (SuSy) and starch phosphorylase (SP) and decreased the activity of sucrose phosphate synthase (SPS) and adenosine diphosphate glucose pyrophosphorylase (AGPase) in the source. Water stress increased the activity of acid invertase (AI), SuSy and SP but decreased the activity of AGPase in the sink. Potassium modified the balance of enzymes active in sugar and starch metabolism by increasing the activity of AI, SuSy, SPS and SP and significantly decreasing the activity of AGPase, resulting in increase of hexose. Canonical correlational analysis revealed that the carbon conversion rate was mainly affected by the relative rate of conversion of sucrose to fructose and glucose [*p_1_(t)*] and glucose to starch [*p_5m_(t)*]. SuSy and AGPase had the greatest effect on enzyme activity in the fruit; respectively regulated *p*_1_(*t*) and *p*_5m_(*t*).

## Introduction

Carbon metabolism is a basic physiological metabolism in high plants, but its process is complicated (Winter and Huber, 2000). In most plants, assimilate is transported from source to sink in the form of sucrose, and the ability of a plant organ to obtain assimilate depends to a large extent on its sink strength (Seehuber et al., 2011). Sink strength depends on enzyme activity in sucrose metabolism (Lunn and Macrae, 2003; Du et al., 2020). Enzyme activity, which affects the accumulation of sugar in the fruit, is regulated by external factors such as water and nutrients (Patan and Saita, 2015). It has been found that water stress increases the accumulation of hexose and thus improves fruit quality (Terry et al., 2009; Chen et al., 2013). Sugar metabolism is regulated by key enzymes, and water stress affects the activity of key enzymes in the metabolism of carbon assimilates (Mafakheri et al., 2011). Nutrients, such as potassium, promote the conversion and transport of photosynthesis products, which include sugars (Deeken et al., 2002; Walter and Difonzo, 2007). Thus plant respiration, enzyme activity, and sugar metabolism are all influenced by potassium (Philippe et al., 2006; Almeselmani et al., 2009; Zahoor et al., 2017b; Omondi et al., 2019), and so both water and mineral nutrients are key factors in determining carbon allocation (Reynolds and Tuberosa, 2008; Rosa et al., 2009; Witt et al., 2012).

As the “source” organ, the leaf is the only resource depend on for existence in plants (Büchi et al., 1998). Sugars and starch are important carbon metabolites in plants. Sucrose metabolism is critical to a plant’s success because it regulates enzyme activity in photoassimilates transported to sink tissue, thereby affecting the accumulation and type of various sugars in the fruit (Chopra et al., 2005; Jain et al., 2013). The enzymes that catalyze sucrose metabolism are mainly invertase β-fructofuranosidase (EC 3.2.1.26, Inv), sucrose synthase (EC 2.4.1.13, SuSy), and sucrose phosphate synthase (EC 2.4.1.14, SPS) (Schaffer and Petreikov, 1997; Tymowska-Lalanne and Kreis, 1998; Binh et al., 1999; Roitsch and Mari, 2004). They regulate the distribution of carbohydrates in source and sink organs, control the rate of sucrose absorption, and govern the storage of sucrose and hexose (Ruan, 2014). Starch metabolism is an accurate, systematic and complex process that includes starch synthesis and conversion. Starch phosphorylase (EC 2.4.1.1, SP) and ADP-glucose pyrophosphorylase (EC 2.7.7.27, AGPase) are the principal enzymes in starch metabolism (Vardy et al., 2002; Rathore et al., 2009; Subasinghe et al., 2014).

Carbon metabolism had been well studied. The SUGAR model was developed to represent the partitioning of carbon in peaches and to calculate the rates of carbon conversion; the model has been improved since its introduction (Génard and Souty, 1996). Subsequently, the model was used to predict the variation of different sugar concentrations in peach fruit with development and relative fruit growth rate under different environmental or management conditions (e.g., water deficit, thinning and different light interception) (Génard et al., 2003) or to simulate the sugar accumulation process in grape during the veraison-maturation stage (Dai et al., 2009). On this basis, Prudent et al. (2011) combined the two variables of genotype and sink-source ratio to describe the sugar accumulation in tomato fruits from the perspective of physiology and ecology, to provide a basis for understanding the physiological process of sugar accumulation in tomato.

However, there are few studies of how enzymes regulate carbon allocation in tomato under different water and nutrient supply condition, or of the relationship between enzymes and the carbon conversion rate. We analyzed and compared changes in enzyme activity in sucrose metabolism and starch metabolism while controlling water stress and potassium supply. We also investigated the relationship between enzymes and carbon conversion to identify the most important factors. We verified our model of the effects of water and potassium on enzyme activity and sugar metabolism, thus providing a theoretical basis for improving fruit quality by controlling sugar accumulation in the fruit.

## Materials and Methods

### Plant Materials and Growth Conditions

The experiments were conducted in a greenhouse at the Shiyanghe Experimental Station (37°52′N, 102°50′E, 1581 m elevation), Gansu Province, Northwest China, from April to August 2017. The greenhouse, 76 m × 8 m, was a steel frame construction covered with 0.2 mm thick polyethylene. A ventilation system on the roof controlled the interior daytime temperature in summer. Temperatures in the greenhouse from April-August 2017 ranged from 14.83 to 30.98°C and humidity from 25.98 to 91.42 RH. The research plant was an indeterminate pink tomato (*Lycopersicon esculentum* Miller cv. Jinpeng 11), a cultivar that is commonly planted by local farmers. Therefore, after the fifth fruit trusses appearance, the plants were pruned by removing the apex to stop the vegetative growth.

Plants in all treatments were fully irrigated at the seedling stage to ensure plant survival. At the third to fourth leaf stage, the single seedlings were transplanted into each plastic containers (top diameter 33 cm, bottom diameter 25 cm, depth 28 cm) and the container was buried in the ground up to its top edge to maintain a soil temperature in the container similar to that in the surrounding field. Cheesecloth and 1 kg of small gravel were packed at the bottom of each container to prevent soil loss, and the containers were filled with 17 kg of air-dried sandy loam soil (particle size < 5 mm) with bulk density 1.3 ± 0.5 g⋅cm^–3^. Planting was carried out in a single hole and single plant, with a row spacing of 80cm and a plant spacing of 60cm at the experimental site, with one drip irrigation belt controlling one crop row. The plants in each treatment were arranged in six north–south rows of 10 plants, a total of 240 plants. For each treatment, flowers of the first and fourth trusses in ten plants were marked with their pollination date. Due to the small north-south span in the greenhouse, the crops planted close to the underside of the vents and the edges of the greenhouse film are affected by the boundary effect to a certain extent, the experimental sites should be as far away from the inner greenhouse boundary as possible. In order to measure the fruit various indicators more accurately and to avoid errors in the results due to sampling, random sampling was concentrated in 2nd to 8th row. The experiment layout and sampling diagram were shown in Supplementary Figure 1. The basic physical properties of the soil were volumetric field capacity 0.258 (cm^3^⋅cm^–3^), saturated paste extract electrical conductivity 0.205 dS⋅m^–1^, available potassium 88 mg⋅kg^–1^, and pH 7.96.

During entire growth period, the tomato growth stage was divided into flowering and fruit-bearing stage (stage I: 2017-05-14–2017-06-15), fruit-swelling stage (stage II: 2017-06-16–2017-07-13), fruit maturation stage (stage III: 2017-07-14–2017-08-15). These three growth stages represent the stages of cell division (0–15 days after anthesis), cell expansion (15–48 days after anthesis) and maturation (more than 48 days after anthesis) of tomato fruit, according to Guichard et al. (2001) and Ripoll et al. (2015).

### Treatments

Two levels of irrigation, full irrigation (W) and deficit irrigation (W/2) in four water treatments were created: CK (irrigation quantity W in every stage), T_1_ (stage I: W/2), T_2_ (stage II: W/2), T_3_ (stage III: W/2) and the full irrigation quantity W in the other stages in the experiment. Each water treatments was equally divided into 2 subgroups: potassium addition (K_1_) and without potassium (K_0_), the additional potassium treatment was the same for all treatments. Plants that were treated with potassium were identified as a subgroup by appending K to the group label: plants in group CK that were treated with potassium were identified as CKK and plants in the treatment group Ti that were treated with potassium were identified as TiK. The amount of potassium to be applied for optimum fruit development was determined from previous literature to be 0.46 g/kg (K_2_O:soil) per application (Han et al., 2012; Feng et al., 2017). Thus half of the plants was treated with a total of 15.64 g K_2_O in application twice (Table 1).

**TABLE 1:** Details of irrigation amount and potassium application in different water and potassium treatments in the three stages.

**Treatments**	**Irrigation amount (mm)**				**Potassium amount (g K_2_O)**	
	**Stage I**	**Stage II**	**Stage III**	**Total**	**Date:06/01**	**Date:06/05**
T_1_	30.33	114.92	91.64	254.97	0	0
T_2_	60.66	57.46	91.64	227.84	0	0
T_3_	60.66	114.92	45.82	239.48	0	0
CK	60.66	114.92	91.64	285.30	0	0
T_1_K	30.33	114.92	91.64	254.97	7.82	7.82
T_2_K	60.66	57.46	91.64	227.84	7.82	7.82
T_3_K	60.66	114.92	45.82	239.48	7.82	7.82
CKK	60.66	114.92	91.64	285.30	7.82	7.82

**Different water treatments (i.e., T_1_, T_2_, T_3_, and CK) and potassium treatments (i.e., T_1_K, T_2_K, T_3_K, and CKK) at three stages was listed. Water deficit was divided into three stages, namely Stage I: from 05/14 to 06/15, Stage II: from 06/16 to 07/13, Stage III: from 07/14 to 08/15. The same potassium amount was applied to the flowering and fruit-bearing stage (June 1 and 5), a total of 15.64 g K_2_O.**

### Test Items and Methods

#### Irrigation Amount

A 5TE sensor (Decagon Devices, Inc., United States) was installed at 15 cm depth in three randomly selected containers in every treatment to measure soil water content (SWC; cm^3^/cm^3^). The data were collected every 30 min by an EM50 data logger (Decagon Devices, Inc., United States). The sensors were calibrated gravimetrically using sensor-measured data for volumetric water content. When the water content in the containers decreased to 70% of field capacity θ_*f*_ (Agbenin and Tiessen, 1995), which was determined using the cutting ring method (Hu et al., 2011), the pots were irrigated to about 95% of field capacity. The amount of irrigation water was calculated using the equation:

(1) $$W=\left(\theta_{t1}-\theta_{t2}\right)\times V\;$$

where *W* (cm^3^) is the irrigation amount; θ_*t*__1_ and θ_*t*__2_ (cm^3^⋅cm^–3^) are, respectively, the upper limits of soil water content and the measured soil water content before irrigation; and *V* (cm^3^) is the pot soil volume. To prevent irrigation water leakage from the containers, irrigation occurred over a short period, and the irrigation quantity did not exceed field capacity. Irrigation quantities and potassium amounts applied during all growth stages are given in Table 1.

#### Index Measurement

Fruits were picked at 34 days after anthesis (DAA) from the first truss; 37, 48, and 57 DAA from the second truss; 58 and 65 DAA from the third truss; and 66 and 73 DAA from the fourth truss; the sampling of the leaves corresponds to the first leaf under each truss of fruits that has been picked, and each treatment was replicated three times. After picking, the fruit and leaves were quickly transferred to the laboratory, washed in distilled water and left to dry in a cool place. The fruit was cut open and a portion with pulp was weighed, then ground and mixed in a juicer for the determination of glucose, fructose, sucrose, starch content and enzyme activities related to sucrose and starch metabolism. Sugar content and related enzyme activities were measured every 5–7 days in fruit, and the whole growth stage was measured 8 times in total.

#### Sugar and Potassium Determination

Soluble sugars were extracted using the procedures described in Gomez et al. (2002) and assayed by HPLC analysis. Starch content was determined enzymatically using the method described in Gomez et al. (2003). The potassium content was determined by employing atomic absorption spectrophotometry (Xue et al., 2006).

#### Enzyme Extraction and Assays

SPS, SuSy and AI: leaf and fruit samples (0.2 g fresh weight) and 10 mL of extraction buffer (50 mM Hepes-NaOH, pH 7.5, 10 mM MgCl_2_, 2 mM EDTA, 5 Mm DTT, 2% (w/v) PVP) were ground into a homogenate in an ice bath. The samples were centrifuged subsequently at 12,000 × *g* for 20 min at 4 °C. The supernatant was gradually added with ammonium sulfate to 80% saturation and then centrifuged at 12,000 × *g* for 20 min at 4°C. The supernatant was discarded and the precipitate was dissolved with 3 mL of extraction buffer and then dialyzed for 20 h with 10-fold dilution of extraction buffer (without PVPP).

SPS activity was determined by the method of Keller and Ludlow (1993) with minor modifications. 50 μL enzyme solution was added to 50 μL 100 mM Hepes-NaOH buffer, 20 μL 50 mM MgCl_2_, 20 μL 100 mM UDPG, and 20 μL 100 mM fructose 6-phosphate. After 30 min, the reaction was terminated by the addition of 200 μL 40% NaOH solution, followed by 1.5 mL 30% HCl and 0.5 mL 1% resorcinol to determine the sucrose production, the unit of enzyme activity was expressed as μmol Suc⋅g^–1^⋅FW⋅h^–1^. SuSy activity (synthetic direction) was determined by replacing fructose 6-phosphate with fructose in the same way as SPS activity, the unit of enzyme activity was expressed as μmol Suc⋅g^–1^⋅FW⋅h^–1^. The AI activity was determined by the methods of Merlo and Passera (1991), 0.2 mL enzyme solution was added into 0.8 mL reaction solution (pH 4.8 0.1M Na_2_HPO_4_-0.1M sodium citrate, 0.1M sucrose), and reacted at 37 °C for 30min, the unit of enzyme activity was expressed as μmol Glu⋅g^–1^⋅FW⋅h^–1^.

SP: 0.3 g of fresh samples was taken into a pre-cooled mortar, enzyme extraction medium was added at w:v = 1:5 and ground into a homogenate in a rapid ice bath. The grindings were filtered through 4 layers of gauze, centrifuged at 4°C for 10 min at 12,000 × *g* and the supernatant was poured out as the crude enzyme solution. SP was determined according to the method of Merlo and Passera (1991), the reaction was started by adding 0.2 mL of reaction medium, 0.65 mL of distilled water, 0.05 mL of enzyme solution and finally 0.1 mL of Glu-1-P in a water bath at 30°C for 10 min. 0.5 mL of 5% TCA was added to terminate the reaction. For CK, TCA was added before the enzyme solution, and the other steps were performed as above. Centrifuged the reacted solution at 4,000 × *g* for 10 min to discard the precipitate, taken the supernatant for inorganic phosphorus determination. 0.3 mL supernatant and 2.7 mL of distilled water were added into the test tube, and then 3 mL of phosphate reagent was added. The liquid was shaken well and kept warm in 45°C water bath for 25 min. The absorbance was measured at 660nm wavelength and calculated, the unit of enzyme activity was expressed as μg Pi⋅g^–1^⋅FW⋅min^–1^.

AGPase: a fresh sample of 0.3 g was peeled and placed in a mortar after an ice bath and ground into a homogenate by adding 3 mL of extraction medium (100 mM Hepes-NaOH with pH 7.6, 2 mM EDTA, 5 mM DTT, 8 mM MgCl_2_, 12.5% (v/v) propanetriol and 5% (w/v) PVP), and then centrifuged at 12,000 × *g* for 10 min. Two milliliter of supernatant was taken into a 5 mL centrifuge tube and used for enzyme activity determination. AGPase was determined according to the method of Fernie et al. (2001), 100 μL 5 mM ADPG, 50 μL 50 mM MgCl_2_, 100 μL buffer, 50 μL enzyme extract, 100 μL 20 mM PPi was added to start the reaction for 15 min, and the reaction was terminated by boiling water bath for 1 min. Cool, add 100 μL 6 mM NADP^+^, 1.5U phosphoglucose metatase, 50 μL 5UL^–1^ 6-P-G dehydrogenase, 0.3 mL buffer, total volume 1.5 mL, react at 30°C for 10min then colorimetric at 340 nm, use 1-P-G for standard curve, the unit of enzyme activity was expressed as nmol Glu⋅g^–1^⋅FW⋅min^–1^.

#### Related Model Parameters and Metabolism

**FIGURE 1:**
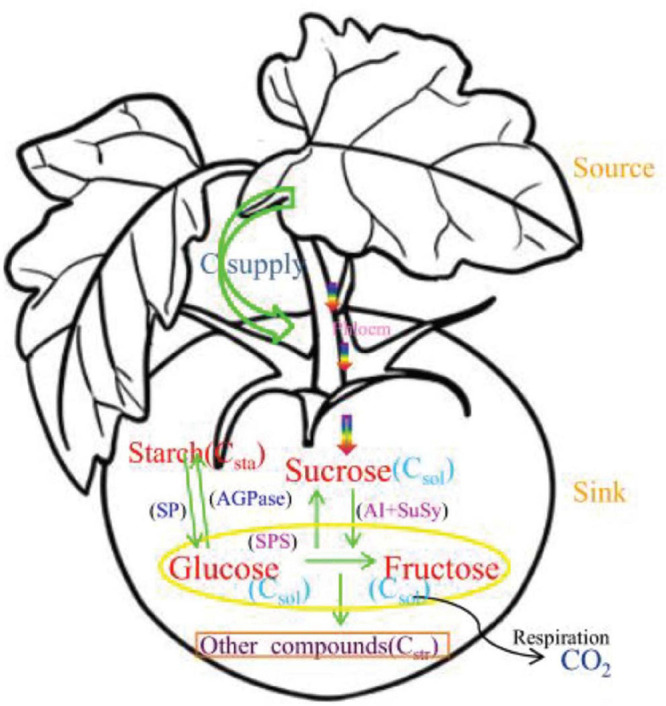
Carbon metabolism and related enzymes in tomato fruit. Arrows represent carbon flows, and the parameters *p*_1_(*t*), *p*_2_(*t*), *p*_3_(*t*), *p*_4_(*t*), *p*_4_*_*m*_*(*t*), and *p*_5_*_*m*_*(*t*) are the relative rates of carbon conversion for fructose, glucose, sucrose, starch and other compounds. The source is the leaf, which supplies carbon; the sink is the fruit, to which carbon is transported. Green arrows show the interconversion of different forms of carbon catalyzed by enzymes in the fruit. The yellow ellipse contains glucose and fructose, which are the main sugars in the tomato, orange rectangle denotes other carbon-containing compounds and the black curved line indicates carbon lost through respiration.

The main physiological processes of carbon metabolism in tomato plants are shown in Figure 1. Sucrose is converted into glucose and fructose by sucrose invertase and sucrose synthase in fruits (María et al., 2000). Glucose and fructose are converted to sucrose by sucrose-phosphate synthase (Galtier et al., 1993). Glucose and fructose are also converted into each other (Makkee et al., 1984). Starch is synthesized from glucose by adenosine diphosphate glucose pyrophosphorylase (Schaffer and Petreikov, 1997); starch is also converted to glucose by amylase and inorganic pyrophosphatase (Nettancourt and Gösta, 1968). Starch compartment in tomato fruit is explicitly described in detail in Chen (2016); which followed principle of carbon balance (Walker and Thornley, 1977). Carbon allocation and transformation in fruit is closely related to the activity of metabolic enzymes during growth and development (Sharkey et al., 1991; Coleman et al., 2009). The carbon conversion rate can be calculated using Wu’s equations for peach fruit (Wu et al., 2012). The equations that describe diurnal carbon variation are the following.

(2) $$\frac{dC_{su\text{c}}}{dt}=p_{0}\frac{dC_{sup}}{dt}-p_{1}C_{suc}+\frac{p_{2}}{2}\left(C_{glu}+C_{fru}\right)$$

$$\frac{dC_{glu}}{dt}=\frac{p_{1}}{2}C_{suc}+p_{4m}C_{fru}+p_{5}C_{sta}$$

(3) $$-\frac{C_{glu}}{C_{glu}+C_{fru}}\frac{dC_{rep}}{dt}-\left(\frac{p_{2}+p_{3}}{2}+p_{5m}+p_{4}\right)C_{glu}$$

$$\frac{dC_{fru}}{dt}=\frac{p_{1}}{2}C_{suc}+p_{4}C_{glu}-\frac{C_{fru}}{C_{glu}+C_{fru}}\frac{dC_{rep}}{dt}$$

(4) $$-\left(\frac{p_{2}+p_{3}}{2}+p_{4m}\right)C_{fru}$$

(5) $$\frac{dC_{sta}}{dt}=p_{5m}C_{glu}-p_{5}C_{sta}$$

(6) $$\frac{dC_{str}}{dt}=\frac{p_{3}}{2}\left(C_{glu}+C_{fru}\right)$$

$$\frac{dC_{DW}}{dt}=\frac{dC_{suc}}{dt}+\frac{dC_{glu}}{dt}+\frac{dC_{fru}}{dt}$$

(7) $$+\frac{dC_{sta}}{dt}+\frac{dC_{str}}{dt}=\frac{dC_{sup}}{dt}-\frac{dC_{rep}}{dt}$$

(8) $$\frac{dC_{DW}}{dt}=c_{DW}\frac{dDW}{dt}$$

(9) $$\frac{dC_{rep}}{dt}=q_{g}\frac{dDW}{dt}+q_{m}DWQ_{10}^{\left(t-20\right)/10}$$

The parameters in the equations are defined in Table 2. Parameter values were found using the nls() and optim() functions in the computer language R. The process of solving for the parameters has been explained in detail by Luo et al. (2020) and is not shown here. Previous research have shown that glucose and fructose content in tomato fruits are almost equal (Islam et al., 1996; Levin et al., 2000; Qi et al., 2005), thus the parameters *p*_4_(*t*) and *p*_4m_(*t*) between glucose and fructose can be assumed to be a constant. The parameter *p*_5_(*t*) was considered to be a constant in previous studies (Nguyen-Quoc and Foyer, 2001; Chen et al., 2020) and is not discussed further. In this study, we focus on the relationship between enzymes active in sucrose and starch metabolism and the carbon conversion rates *p*_1_(*t*), *p*_2_(*t*), *p*_3_(*t*), and *p*_5_*_*m*_*(*t*).

**TABLE 2:** Definitions of carbon allocation and conversion parameters.

**Parameter**	**Definition**	**Unit**
*C*_*sup*_	Carbon imported into the fruit from the phloem	g C
*C*_*rep*_	Carbon consumed through respiration	g C
*C*_*suc*_	The amount of carbon in the form of sucrose	g C
*C*_*glu*_	The amount of carbon in the form of glucose	g C
*C*_*fru*_	The amount of carbon in the form of fructose	g C
*C*_*sta*_	The amount of carbon in the form of starch	g C
*C*_*str*_	The amount of carbon in the form of other structural compounds	g C
*FW*	Fresh mass of the fruit	g
*DW*	Dry mass of the fruit	g
*c*_*DW*_	The carbon amount of 1 g of dry mass, is 0.44	g C/g DW
*q*_*g*_	The growth respiration coefficient, is 0.088	g C/g DW
*q*_*m*_	The maintenance respiration coefficient at 20 °C, is 0.000 168	g C/g DW
*Q*_10_	The temperature ratio of maintenance respiration, is 1.4	Dimensionless
*T*	Temperature	°C
*dC*_*sup*_/*dt*	The daily carbon flows into the fruit imported from the phloem	g C hr. ^–1^
*dC*_*rep*_/*dt*	The daily carbon flows out of the fruit by respiration	g C hr. ^–1^
*p*_0_(*t*)	Imported from the phloem in the form of sucrose and is assumed to be 1	dimensionless
*p*_1_(*t*)	The relative rates of carbon conversion from daily net sucrose to glucose and fructose	d^–1^
*p*_2_(*t*)	The relative rates of carbon conversion from daily net glucose and fructose to sucrose	d^–1^
*p*_3_(*t*)	The relative rates of carbon conversion from daily net glucose and fructose to other compounds	d^–1^
*p*_4_(*t*)	The relative rates of carbon conversion from daily net glucose to fructose	d^–1^
*p*_4m_(*t*)	The relative rates of carbon conversion from daily net fructose to glucose	d^–1^
*p*_5_(*t*)	The relative rates of carbon conversion from daily net starch to glucose	d^–1^
*p*_5m_(*t*)	The relative rates of carbon conversion from daily net glucose to starch	d^–1^

#### Canonical Correlation Analysis of Enzyme and Carbon Conversion Rate

The vector composed of the parameters for the carbon conversion rate and enzyme activity measured in the fruit was used for canonical correlation analysis. The results were calculated according to Equations (10) and (11).

(10) $$U_{1}=0.044AI+0.987SuSy-0.280SPS+0.358SP-0.851AGPase$$

(11) $$V_{1}=0.941p_{1}-0.279p_{2}+0.512p_{3}-0.940p_{5m}$$

where *U*_1_ represents the linear combination of various enzyme activities in the fruit, and *V*_1_ represents the linear combination of the rates of carbon conversion. The significance of canonical variables is determined mainly by the variables with greater load.

#### Statistical Analysis

Three-way analysis of variance was performed using R studio version 3.6.1 (Robert, 2016) to evaluate the individual effects, and any interactive effects, of the three factors *irrigation*, *potassium*, and *growth stage* on source–sink enzyme activity (Tables 3, 5). Mean values were used for water treatments (shown by different letters), and the least significant difference (LSD) and multiple range tests were used to calculate differences between treatments at confidence level *P* < 0.05. Canonical correlational analysis, Spearman correlation analysis, multiple linear regression, nonlinear regression and the Kruskal–Wallis test were carried out using R, and the ggplot2-based plots were drawn using R packages ggpubr, ggthemes and PerformanceAnalytics (Alboukadel, 2017).

**TABLE 3:** Three-way analysis of variance of leaves SuSy, SPS, SP, and AGPase were performed to identify individual and interactive effects of water (2 levels: water deficit and CK), potassium (2 levels: K_0_, K_1_) and growth stage (2 levels: stage II and stage III) during all growth stages.

**Statistics**	**Treatment**	**SuSy (μmol Suc⋅g**^–^**^1^⋅FW⋅min**^–^**^1^)**	**SPS (μmol Suc⋅g**^–^**^1^⋅FW⋅h**^–^**^1^)**	**SP (μg Pi⋅g**^–^**^1^⋅FW⋅min**^–^**^1^)**	**AGPase (nmol Glu⋅g**^–^**^1^⋅FW⋅min**^–^**^1^)**
**LSD**	**Water level (W)**				
	WD	20.380 ± 3.536a	13.174 ± 3.148b	237.106 ± 74.242a	92.918 ± 50.444b
	CK	17.476 ± 2.612b	19.326 ± 2.100a	201.803 ± 51.711b	168.500 ± 80.409a
	**Potassium level (P)**				
	K_0_	19.654 ± 3.549b	14.712 ± 3.956b	228.280 ± 70.732b	111.813 ± 67.449a
	K_1_	23.136 ± 3.732a	19.009 ± 3.823a	271.214 ± 76.075a	86.510 ± 52.903b
	**Growth stage (S)**				
	Stage II	19.068 ± 3.477b	18.037 ± 3.968a	269.224 ± 82.147a	62.681 ± 38.855b
	Stage III	23.723 ± 3.100a	15.684 ± 4.588b	230.27 ± 64.861b	135.642 ± 58.925a
**ANOVA**	W	89.066***	207.213***	20.383***	69.676***
	P	138.364***	138.084***	18.193***	3.659*
	S	323.022***	29.761***	0.107ns	73.318***
	W × P	0.890ns	0.031ns	2.183ns	3.218*
	W × S	0.475ns	26.484***	1.436ns	17.550***
	P × S	0.001ns	0.135ns	2.638ns	0.559ns
	W × P × S	1.494ns	0.403ns	0.021ns	0.787ns
	Residuals	4.2	6.4	4864	2000

The letters following the mean values of water treatments indicate significant differences at P < 0.05 using the LSD method. SuSy, sucrose synthase; SPS, sucrose phosphate synthase; SP, sucrose phosphorylase; AGPase, adenosine glucose pyrophosphorylase. Water treatments: the values shown are mean values for the two water treatments in leaves, deficit irrigation and full irrigation, and ± denotes standard deviation. Potassium treatments: with potassium (K_1_) and without potassium (K_0_) in leaves. Mean values in water and potassium treatments showed significant differences at P < 0.05. *** denotes significant difference at P < 0.001; * denotes significant difference at P < 0.05; ns denotes no significant difference.

## Results

### Activity of Metabolic Enzymes Related to Source Leaves

**FIGURE 2:**
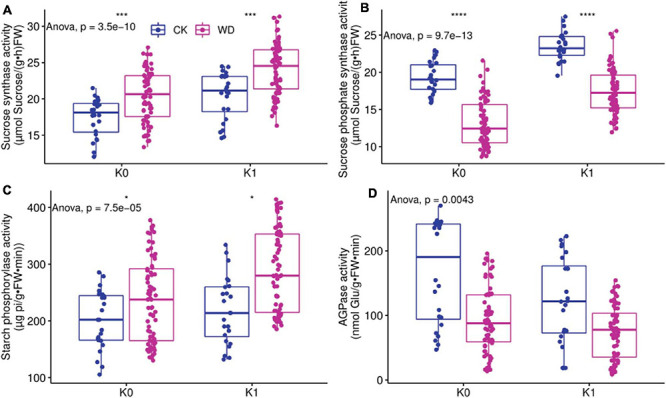
The sucrose synthase (SuSy), sucrose phosphate synthase (SPS), starch phosphorylase (SP), and starch synthase (AGPase) activity in source leaf. Water include 2 level: water deficit (WD) and CK, and potassium level contain without potassium (K_0_) and with potassium (K_1_). **(A)** SuSy activity in leaf under deficit irrigation and potassium application condition. **(B)** SPS activity in leaf under deficit irrigation and potassium application condition. **(C)** SP activity in leaf under deficit irrigation and potassium application condition. **(D)** AGPase activity in leaf under deficit irrigation and potassium application condition. * denotes significant difference at *P* < 0.05, *** denotes significant difference at *P* < 0.001, **** denotes significant difference at *P* < 0.0001.

Figure 2 showed the activity of sucrose synthase (SuSy), sucrose phosphate synthase (SPS), starch phosphorylase (SP), and adenosine glucose pyrophosphorylase (AGPase) in tomato leaves for different treatments; K_0_ denotes *without potassium*, and K_1_ denotes *with potassium*. CK denotes *full irrigation*, and WD denotes *water deficit* treatments (T_1_, T_2_, and T_3_).

The effects of water deficit and potassium on SuSy activity in leaves were considerable (Table 3). SuSy activity was greater in water deficit treatments (WD) than in full irrigation (CK) regardless of whether potassium was applied or not; regardless of water deficit or full irrigation, the SuSy activity of potassium treatment (K_1_) was significantly greater than without potassium treatment (K_0_), with was greatest in the water deficit and potassium treatments but least in CK.

The SPS activity of WD was significantly less than CK regardless of whether potassium was applied or not; whether full irrigation or water deficit conditions, K_1_ was greater than K_0_. The interactive effect of water deficit and stage had a significant effect on SPS, and WD was the least (Figure 2B).

The effects of water deficit and potassium application on SP activity were very noticeable (Table 3). Whether potassium was applied or not, the SP activity of WD was greater than CK; whether full irrigation or water deficit conditions, K_1_ was significantly greater than K_0_, of which water deficit and potassium condition was the greatest, and CK was the lowest (Figure 2C).

The effect of water deficit and potassium application on AGPase activity was highly significant, the interaction of water and potassium application had a significant impact on AGPase, and the interactive effect of water deficit and stage was considerable (Table 3). AGPase activity for WD was significantly less than for CK whether or not potassium was applied; AGPase activity was less for K_1_ than for K_0_ both under full irrigation or water deficit conditions and greatest in CK (Figure 2D).

### Carbon Flux in the Sink Under Water and Potassium Supply

**FIGURE 3:**
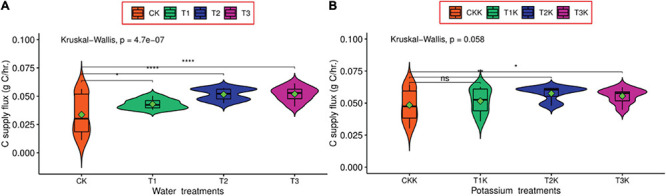
Diurnal variation of carbon supply flux between K_0_ and K_1_ treatments during the all growth stages. K_0_ indicates no potassium and K_1_ indicates potassium addition. The Wilcoxon signed rank test was used, ns denotes no significant difference, * denotes significant difference at the confidence level *P* < 0.05, *** denotes significant difference at the confidence level *P* < 0.001, **** denotes significant difference at P < 0.0001. **(A)** Carbon supply for different water treatments. **(B)** Carbon supply for different water and potassium treatments.

According to equation (7), the *dC_*sup*_/dt* was the carbon import to the fruit through the phloem, including the total carbon amount of dry matter and the carbon consumption through respiration, which can directly reflect the carbon flows into the fruit; and equation (9) showed that *dC_*rep*_/dt* was only related to dry matter, and the others are constants. Carbon flux (*dC_*sup*_/dt*) for the water deficit treatments (T_1_, T_2_ and T_3_) was greater than CK, compared with CK, T_1_ was significantly difference, both T_2_ and T_3_ were an extremely significant under water treatments (Figure 3A). However, in the potassium treatments (T_1_K, T_2_K, T_3_K, and CKK), the difference between T_1_K and CKK was not significant, T_2_K and CKK was very significant, T_3_K and CKK was significant (Figure 3B), in addition, all potassium treatments were greater than CK.

### Sucrose-Metabolizing Enzyme Activity in the Sink

**FIGURE 4:**
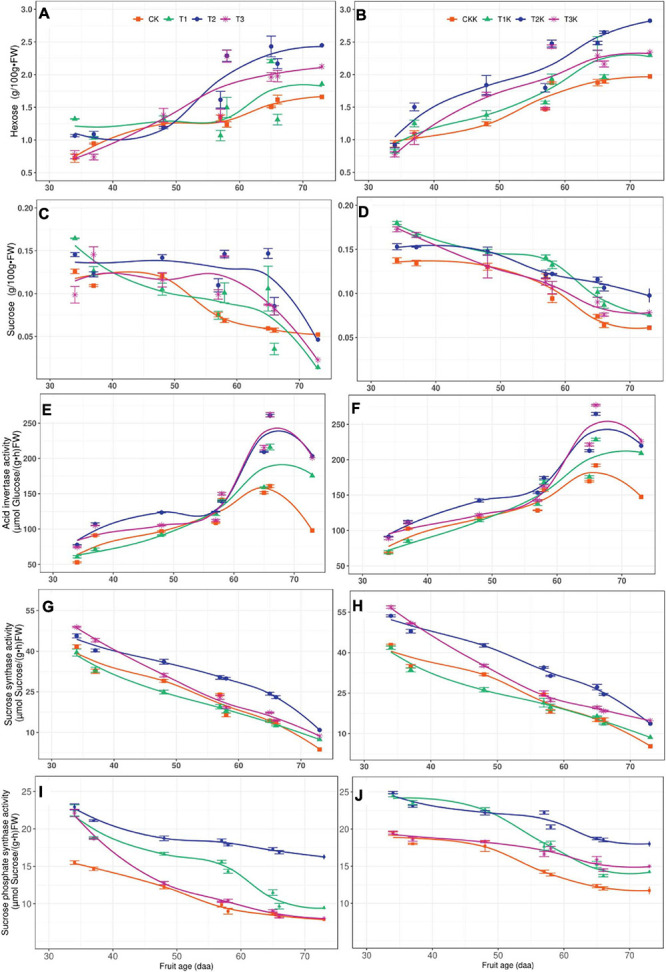
Related sugars and sucrose metabolic enzymes activity in fruit. Measured hexose (points), sucrose (points), acid invertase (points), sucrose synthase (point), sucrose phosphate synthase (point) and their corresponding fitted curves (lines) for the different water and potassium treatments as a function of days after anthesis (DAA). **(A)** Variation of Hexose with fruit age for different water treatments. **(B)** Variation of Hexose with fruit age for different water and potassium treatments. **(C)** Variation of Sucrose with fruit age for different water treatments. **(D)** Variation of Sucrose with fruit age for different water and potassium treatments. **(E)** Variation of AI activity with fruit age for different water treatments. **(F)** Variation of AI activity with fruit age for different water and potassium treatments. **(G)** Variation of SuSy activity with fruit age for different water treatments. **(H)** Variation of SuSy activity with fruit age for different water and potassium treatments. **(I)** Variation of SPS activity with fruit age for different water treatments. **(J)** Variation of SPS activity with fruit age for different water and potassium treatments.

Figure 1 showed that the enzymes related to sucrose metabolism were SuSy, AI and SPS, and the sugars involved were hexose (glucose+fructose) and sucrose, sugars and enzymes activity over the entire fruit growth period were shown in Figure 4. Hexose increased with the fruit age, of which T_2_ was the highest and CK was the lowest, the difference between water treatments was significant. Compared with CK, T_1_, T_2_, and T_3_ increased by 13.18, 44.96, and 22.48%, respectively. The sucrose tended to decrease gradually with fruit age, but the range of variation was not large.

Mean sucrose was ordered by treatment T_2_ > T_3_ > T_1_ > CK and water deficit had a significant on sucrose. The trends in sucrose for potassium treatments were the same as for the water treatments and potassium application had a significant effect on sucrose. It was found that both hexose and sucrose were greater for K_1_ (T_1_K, T_2_K, T_3_K, and CKK) than for K_0_ (T_1_, T_2_, T_3_, and CK) (Table 4).

**TABLE 4:** Three-way analysis of variance of fruits Hexose, Sucrose, Glucose, Fructose and Strach were performed to identify individual and interactive effects of water (4 levels: T_1_,T_2_,T_3_, and CK), potassium (2 levels: K_0_, K_1_) and growth stage (2 levels: stage II and stage III) on the tomato fruits.

**Treatment**	**Hexose (g/100 g⋅FW)**	**Sucrose (g/100 g⋅FW)**	**Glucose (g/100 g⋅FW)**	**Fructose (g/100 g⋅FW)**	**Starch (g/100 g⋅FW)**
**Water treatment (W)**					
T_1_	1.46 ± 0.20c	0.10 ± 0.05b	0.74 ± 0.20c	0.72 ± 0.20c	0.31 ± 0.13a
T_2_	1.87 ± 0.30a	0.12 ± 0.04a	0.95 ± 0.30a	0.92 ± 0.30a	0.27 ± 0.14a
T_3_	1.58 ± 0.29b	0.10 ± 0.04b	0.80 ± 0.30b	0.78 ± 0.29b	0.22 ± 0.08a
CK	1.29 ± 0.15d	0.08 ± 0.03c	0.65 ± 0.16d	0.64 ± 0.15d	0.19 ± 0.14a
**Potassium treatment (P)**					
K_0_	1.55 ± 0.26b	0.10 ± 0.04b	0.79 ± 0.26b	0.76 ± 0.26b	0.24 ± 0.20a
K_1_	1.77 ± 0.29a	0.12 ± 0.03a	0.89 ± 0.29a	0.88 ± 0.29a	0.23 ± 0.15a
**Growth stage (S)**					
Stage II	1.22 ± 0.16b	0.12 ± 0.03a	0.62 ± 0.16b	0.60 ± 0.16b	0.37 ± 0.15a
Stage III	2.08 ± 0.20a	0.09 ± 0.04b	1.05 ± 0.20a	1.03 ± 0.20a	0.15 ± 0.06b
W	32.083***	6.615***	32.005***	30.002***	2.575*
K	43.294***	17.856***	41.750***	40.10***	0.023ns
S	524.552***	46.098***	519.857***	459.023***	48.184***
W × P	0.213ns	1.463ns	0.242ns	0.203ns	0.327ns
W × S	10.911***	3.483*	10.469***	9.939***	1.952ns
P × S	5.524*	4.732*	4.832*	4.523*	0.140ns
W × P × S	1.474ns	0.299ns	1.418ns	1.211ns	0.657ns
Residuals	0.07	0.001	0.017	0.015	0.026

The letters following the mean values of water treatments indicate significant differences at P < 0.05 using the LSD method. The values shown are the average values in tomato fruits for the four water treatments T_1_, T_2_, T_3_ and CK, in which T_1_, T_2_, and T_3_ were water deficit treatments. Different letters (a, b, c) following the mean values in water treatments indicate significant differences at the P < 0.05 level. Differences due to the treatment were determined by analysis of variance for S, growth stage; W, water effect; P, potassium effect; W × S: interactive effect of water and stage; P × S: interactive effect of potassium and stage; *** denotes significant difference at P < 0.001; * denotes significant difference at P < 0.05; ns denotes no significant difference.

AI activity increased gradually as DAA increased and reached a maximum at 66 DAA, and then began to decrease (Figure 4E). The order of the AI mean was: T_2_ > T_3_ > T_1_ > CK, the AI for the water deficit treatments was higher than for CK. Compared with CK, T_1_, T_2_, and T_3_ increased by 15.07, 38.24, and 36.03%, but the difference between T_2_ and T_3_ was not notable. The results of F-test showed that water and potassium application had significant effect on AI, and the AI activity of Stage III was greater than Stage II. The variation trend of AI in potassium treatments was the same as that of water treatments (Figure 4F).

SuSy activity gradually decreased as DAA increased and reached a very low level at maturity (Figures 4G,H). The mean of SuSy activity under different water conditions was: T_2_ > T_3_ > T_1_ > CK, with water deficit treatments displayed significantly greater than CK. T_1_, T_2_, and T_3_ showed increases of 5.52, 37.12, and 17.88%, respectively, compared to CK. The potassium addition resulted in a very significant change in SuSy activity, SuSy was greater for K_1_ than K_0_ (Table 5).

**TABLE 5:** Three-way analysis of variance of fruits AI, SuSy, SPS, SP, and AGPase, were performed to identify individual and interactive effects of water, potassium and growth stage on the tomato fruits.

**Treatment**	**AI (μmol Glu⋅g**^–^**^1^⋅FW ⋅h**^–^**^1^)**	**SuSy (μmol Suc⋅g**^–^**^1^⋅FW⋅h**^–^**^1^)**	**SPS (μmol Suc⋅g**^–^**^1^⋅FW ⋅h**^–^**^1^)**	**SP (μg Pi⋅g**^–^**^1^⋅FW ⋅min**^–^**^1^)**	**AGPase (nmol Glu⋅g**^–^**^1^⋅FW⋅min**^–^**^1^)**
**Water treatment (W)**					
T_1_	129.59 ± 51.44b	23.14 ± 10.40b	14.82 ± 4.38b	290.84 ± 71.54b	322.70 ± 46.03a
T_2_	155.69 ± 39.21a	30.07 ± 10.47a	18.70 ± 2.19a	302.62 ± 63.89a	315.22 ± 52.94a
T_3_	153.20 ± 53.08a	25.85 ± 13.72b	12.46 ± 1.65c	285.08 ± 53.90b	328.71 ± 54.23a
CK	112.62 ± 34.63c	21.93 ± 11.70c	10.76 ± 2.71d	254.15 ± 46.74c	322.33 ± 37.71a
**Potassium treatment (P)**					
K_0_	137.78 ± 55.32b	24.75 ± 12.00b	14.18 ± 4.15b	283.17 ± 74.67b	322.24 ± 47.98a
K_1_	156.07 ± 54.80a	27.74 ± 13.34a	18.06 ± 3.58a	308.71 ± 71.11a	287.21 ± 49.30b
**Growth stage (S)**					
Stage II	103.54 ± 25.11b	35.85 ± 9.85a	18.25 ± 3.93a	315.86 ± 82.16a	341.99 ± 34.14a
Stage III	190.30 ± 42.42a	16.63 ± 6.55b	13.99 ± 3.61b	279.03 ± 58.39b	267.45 ± 35.33b
W	14.640***	29.920***	218.375***	3.852*	1.396ns
P	6.048*	10.303**	8.352*	6.569*	41.577***
S	266.187***	345.306***	313.410***	9.887*	189.268***
W × P	0.116ns	0.897ns	5.673***	0.276ns	0.575ns
W × S	2.937*	3.792*	26.531***	2.410ns	6.094*
P × S	0.466ns	1.256ns	0.006ns	0.689ns	0.144ns
W × P × S	0.018ns	0.368ns	0.217ns	0.207ns	0.516ns
Residuals	1146	38	2	5176	2581

The letters following the mean values of water treatments indicate significant differences at P < 0.05 using the LSD method. AI, acid invertase; SuSy, sucrose synthase; SPS, sucrose phosphate synthase; SP, sucrose phosphorylase; AGPase, adenosine glucose pyrophosphorylase. Different letters (a, b, c) following the mean values in water treatments indicate significant differences at the P < 0.05 level in tomato fruits. Differences due to the treatment were determined by analysis of variance for S, growth stage; W, water effect; P, potassium effect; W × P, interactive effect of water and potassium; W × S: interactive effect of water and stage. *** denotes significant difference at P < 0.001; ** denotes significant difference at P < 0.01; * denotes significant difference at P < 0.05; ns denotes no significant difference.

SPS activity was greatest at the early stage and tended to decrease gradually with fruit age, but increased slightly at harvest stage, such as 73DAA (Figures 4I,J). The order of the SPS mean was: T_2_ > T_1_ > T_3_ > CK, with significant differences among treatments. Compared with CK, SPS increased by 18.94, 50.08, and 15.80% in T_1_, T_2_, and T_3_, respectively. SPS activity was greater for K_1_ than K_0_, showed that potassium had a great effect on SPS. The SPS activity of Stage II was greater than Stage III (Table 5).

### Starch Metabolism Enzymes in the Sink

**FIGURE 5:**
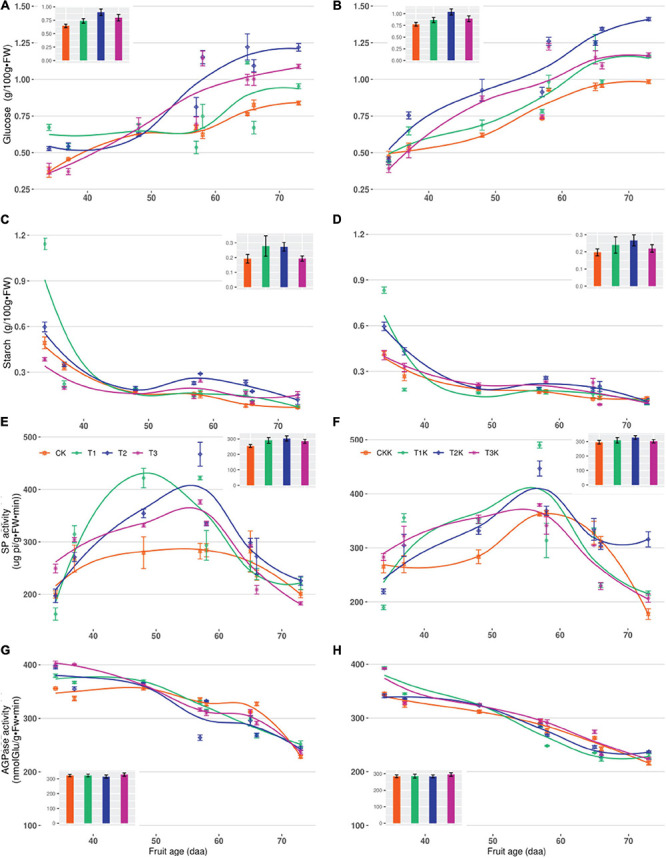
Related sugars and starch metabolic enzymes activity in fruit during the growth stages. Measured glucose (points), starch (points), starch phosphorylase (points), starch synthase (points) and their corresponding fitted curves (lines) for different water and potassium treatments are shown as functions of DAA. The bar chart showed the average value of each treatment. **(A)** Variation of Glucose with fruit age for different water treatments. **(B)** Variation of Glucose with fruit age for different water and potassium treatments. **(C)** Variation of Starch with fruit age for different water treatments. **(D)** Variation of Starch with fruit age for different water and potassium treatments. **(E)** Variation of SP activity with fruit age for different water treatments. **(F)** Variation of SP activity with fruit age for different water and potassium treatments. **(G)** Variation of AGPase activity with fruit age for different water treatments. **(H)** Variation of AGPase activity with fruit age for different water and potassium treatments.

The main enzymes involved in starch synthesis and decomposition were SP and AGPase, and the sugars involved were starch and glucose (Figure 1). Glucose tended to increase gradually with fruit age, reaching a peak at maturity. The order of mean glucose was: T_2_ > T_3_ > T_1_ > CK (Figure 5A), with highly significant differences between different water treatments. Compared with CK, T_1_, T_2_, and T_3_ increasing by 13.85, 46.15, and 23.08%, respectively. Glucose displayed the same variation trend in potassium treatments, and T_2_K was the greatest (Table 4).

Starch tended to decrease gradually with fruit growth and development, reached a minimum at maturity. The order of the mean starch for different water treatments was: T_1_ > T_2_ > T_3_ > CK, however, there was no significant difference among the treatments (Table 4).

The variation of SP and AGPase activity with fruit age (daa) for different treatments during the whole growth stage was shown in Figure 5. SP activity gradually increased with fruit age at the early stage and reached maximum at 48–57 daa, with the T_1_ treatment peaked 453μg pi/g⋅FW⋅min, and then gradually decreased until the harvest stage. The SP mean was: T_2_ > T_1_ > T_3_ > CK from bar chart (Figure 5E). Compared with CK, SP activity in water deficit treatment (T_1_, T_2_, and T_3_) was increased by 14.44, 19.07, and 12.17%, respectively. Change in SP for the potassium treatments showed a parabolic trend, SP increased as fruit age increased, reached a peak at 57 daa and then declined rapidly at during maturation. SP activity was greater for K_1_ than for K_0_, displayed that potassium had a significant effect on SP activity (Table 5).

AGPase activity gradually decreased as fruit age (daa) increased, from the initial value of 410 nmol Glu⋅g^–1^⋅FW⋅min^–1^ to 122 nmol Glu⋅g^–1^⋅FW⋅min^–1^ at maturity (Figure 5G). There was no notable difference in AGPase activity in the water deficit treatments (T_1_, T_2_, and T_3_) compared to CK, and the effect of water on AGPase was not significant. Variation in AGPase activity for potassium treatments (T_1_K, T_2_K, T_3_K, and CKK) showed a similar trend to water treatments (Figure 5H); also AGPase activity was less than for water treatments. This showed that potassium had a notable effect on AGPase activity, which varied significantly at different stages, Stage III was greater than Stage III in AGPase (Table 5).

### Carbon Conversion Rate

**FIGURE 6:**
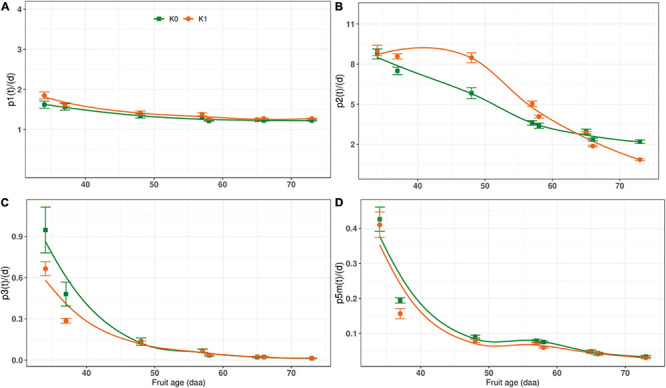
The rate of carbon conversion during the whole growth stage. K_0_ indicates no potassium and K_1_ indicates potassium application. **(A)** The change of carbon conversion rate of sucrose to hexose with fruit age under different potassium condition. **(B)** The change of carbon conversion rate of hexose to sucrose with fruit age under different potassium condition. **(C)** The change of carbon conversion rate of hexose to other compounds with fruit age under different potassium condition. **(D)** The change of carbon conversion rate of glucose to starch with fruit age under different potassium condition.

We discussed the rate of carbon conversion between sugar and starch and other structural carbohydrates in fruit, but did not involve the enzymes associated with glucose and fructose, such as glucose isomerase (GI), and without including *p*_4_(*t*) and *p*_4m_(*t*) (Figure 1). The rate of carbon conversion *p*_1_(*t*), *p*_2_(*t*), *p*_3_(*t*), and *p*_5_*_*m*_*(*t*) were shown in Figure 6. Whether or not potassium was applied, *p*_1_(*t*) did not change significantly during the whole growth stage, and there was almost stable (Figure 6A). The *p*_2_(*t*) gradually decreased with the DAA in the K_0_ treatment, but in the K_1_ treatment, it showed a different trend, increasing first and then decreasing, and was lower than that K_0_ before harvest (Figure 6B). The *p*_3_(*t*) reached a maximum in the early stage and then decreased to near zero at maturity. The *p*_3_(*t*) values of the K_1_ were noticeably less than K_0_ at the early stage (Figure 6C). The *p*_5_*_*m*_*(*t*) reached the peak at early stage, gradually dropped with the DAA, and decreased the minimum at harvest under both K_0_ and K_1_ treatments, the *p*_5_*_*m*_*(*t*) of K_0_ was greater than K_1_ (Figure 6D).

### Correlation and Canonical Correlation Analyses of Enzymes and Parameters

According to Equations (10) and (11), SuSy and AGPase had the greatest load in the first canonical variable *U*_1_, indicating that overall enzyme activity in the fruit is mainly determined by SuSy activity and AGPase activity; *p*_1_(*t*) and *p*_5_*_*m*_*(*t*) have the greatest load in *V*_1_, indicating that the conversion rate is mainly determined by *p*_1_(*t*) and *p*_5_*_*m*_*(*t*). SuSy, AGPase and *p*_1_(*t*), *p*_5_*_*m*_*(*t*) can therefore be used as significance indicators in the analysis of correlations between enzyme activity and the carbon conversion coefficients.

**FIGURE 7:**
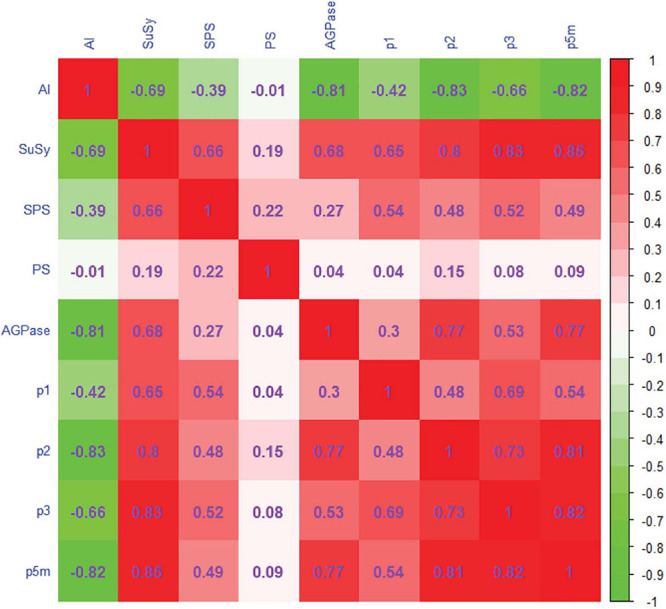
Correlation matrix relating enzyme activity and the rate of carbon conversion in tomatoes. The cell containing the Spearman correlation coefficient (positive or negative) is colored according to the color key (red positive, green negative, white zero). Color intensity increases as the absolute value of the correlation increases.

The Spearman correlation coefficient matrix is shown in Figure 7. The coefficients of correlation between AI and the rates of conversion of fructose and glucose to sucrose [*p*_2_(*t*)], of fructose and glucose to other compounds [*p*_3_(*t*)], and of glucose to starch [*p*_5_*_*m*_*(*t*)] were negative in the range from −0.66 to −0.83. The coefficients of correlation between SuSy and the rates of conversion of sucrose to fructose and glucose, *p*_1_(*t*), *p*_2_(*t*), *p*_3_(*t*), and *p*_5_*_*m*_*(*t*), were in the range 0.65–0.85, which shows significant positive correlations. SPS showed significant weak positive correlations with *p*_1_(*t*), *p*_2_(*t*), *p*_3_(*t*), and *p*_5_*_*m*_*(*t*); the coefficients were in the range 0.48–0.54. SP was not correlated with the rate of carbon conversion. The coefficients of correlation between AGPase and *p*_2_(*t*) and *p*_5_*_*m*_*(*t*) were 0.77, showing significant positive correlations.

## Discussion

Carbon metabolism is closely related to plant growth. Carbohydrates transported from the source (leaves) provide metabolic substrates for fruit growth and non-photosynthetic tissue maintenance (Osorio et al., 2014), and the types of carbohydrate in the sink and their quantities determine fruit quality (Jorquera-Fontena et al., 2018). Sucrose is the principal carbohydrate assimilate in the source. Source loading and leaf metabolism, as well as assimilate transport, are closely related to enzyme activity in the source and sink tissues and significantly affect sugar accumulation in the fruit (Sachdeva et al., 2003). External environmental factors (such as: light, temperature, water, mineral nutrients) also influence carbon metabolism (Merlo and Passera, 1991; Hanson and Roje, 2001).

Sugar is first synthesized in plant mesophyll cells, and transported to the companion cell, and then transported to the phloem via the sucrose transporter, and transported to the sink tissue, thereby regulating the activity of enzymes to promote plant growth (Kühn and Grof, 2010). Therefore, sugar transporters play an important role in sugar transport, such as sucrose transporters (SUT) and hexose and sucrose transporters (SWEET) proteins, which are involved in the transport and distribution of photosynthates, thus regulating physiological processes such as plant response to environmental stresses and seed formation and development (Rae and Grof, 2005).

Sucrose transporter is not only precisely regulated at the transcription and protein level, but also regulated by several external environmental factors, such as light, circadian rhythm, and stress conditions, the presence of which modulates the expression of sucrose transporters (Davies et al., 1999). For example, drought can cause an increase in the phytohormone ABA, which regulates the gene expression of sucrose transporters, thereby enhancing the sensitivity to drought resistance (Yoshida et al., 2010). The transport activity of sucrose transporters gene expression is blocked by sucrose, for example, *OsSUT*1 expression is enhanced when endogenous sugar levels are increased (Matsukura et al., 2000), and there is bound to be a correlation with sucrose transporters as potassium can enhance transport efficiency of the phloem, increase carbon supply (Figure 3) and promote protein synthesis. In addition, almost 90–97% of the total transpiration occurs through the stomata. It is well known that under drought stress conditions, potassium regulates stomatal opening and helps plants adapt to water deficits (Hasanuzzaman et al., 2018), and enhance the cellular osmotic regulation, which in turn alters carbon allocation. Therefore, it is easy to find that the activities of SuSy, SPS, and SP were greater in leaves under potassium application condition, which is conducive to soluble sugar accumulation (Table 3).

Carbon metabolism cannot be achieved without the involvement of enzymes. Our results show that in the water deficit treatments, SuSy activity was significantly higher than in CK, and SPS activity was significantly lower than in CK (Figures 2A,B). The results indicate that water stress inhibited sucrose synthesis and intensified hydrolysis, resulting in more hexose being available to participate in osmotic adjustment (Wu et al., 2007). Whether or not water deficit, SPS activity and SuSy activity were both noticeably greater in the potassium treatments than in the non-potassium treatments, with the two enzyme activities increasing by 15.05 and 29.21%, respectively (Table 3), favoring more soluble sugars in the leaves, which is consistent with previous results (Qi et al., 2005). Moreover, the result also indicates that potassium increases the synthesis of SPS and SuSy in leaves (Table 3) and increases the transport of carbohydrates into the fruit (Figure 3).

The regulation of photosynthesized carbon and its distribution between sucrose and starch in the leaves is dependent on the activity of enzymes active in starch metabolism (Schaffer and Petreikov, 1997; Rathore et al., 2009). Our results showed that water deficit increased SP activity by 17.49% and decreased AGPase activity by 44.86% compared with CK, resulting in a blocked starch synthesis and an enhanced decomposition, which was benefit to starch hydrolysis, which was consistent with previous result (Luengwilai et al., 2010). It was increased SP activity by 18.81% and decreased AGPase activity by 22.63% in K_1_ compared to K_0_ (Table 3), indicating that potassium enhanced amylolytic enzyme activity and inhibited starch synthase activity, which was consistent with the findings of Guo et al. (2019).

Carbon allocation changed in response to environmental changes during fruit development (Geigenberger, 2005; Hermans et al., 2006; Zhang et al., 2014). For example, water stress led to changes in plant growth through reduced photosynthesis (Vu et al., 1999) and increased enzyme activity in sucrose metabolism, which altered carbon allocation in the fruit (Reynolds and Tuberosa, 2008; Witt et al., 2012). Mineral nutrients such as potassium increase enzyme activity in sugar metabolism in the fruit, thus increasing sink strength and promoting assimilation transport and dry matter accumulation (Qi et al., 2005; Gerardeaux et al., 2010). The result displayed that water deficit and potassium application could increase carbon import flux in fruit, which were beneficial to carbon accumulation, and *dCsup/dt* were significantly higher than CK (Figure 3). Water and potassium have an important effect on sucrose and starch metabolism, which determine carbon allocation in the fruit, by regulating enzyme activity in Table 4 (Reynolds and Tuberosa, 2008; Rosa et al., 2009; Witt et al., 2012; Jákli et al., 2018).

Acid invertase is important in sucrose metabolism (Tang et al., 1999); it is a biochemical marker that indicates and regulates sink strength in fruit (Sturm, 1999). AI activity remains at a high level during fruit development, which promotes sucrose conversion and hexose accumulation. AI activity has an important effect on assimilate transportation and conversion and maintains the sucrose concentration gradient from source to sink (María et al., 2000). Compared with CK, the AI activity of water deficit treatments (T_1_, T_2_, and T_3_) increased by 15.07, 38.24, and 36.03%, respectively, indicating that water deficit can increase AI activity and promote hexose accumulation (Tables 4, 5), which was the same as the findings of Qi et al. (2005). The study showed that the AI activity increased significantly after potassium application at maturity in tomato fruit (Han et al., 2012) and the results of our research led to similar conclusion, such as K_1_ > K_0_ (Table 5). Both water deficit and potassium individually raised AI activity (Nicole and Paul, 2004; Zahoor et al., 2017a).

Sucrose synthase is an indicator of sink strength. It is important in sink accumulation (Gupta et al., 2001; Sung et al., 2006). SuSy activity is greater in the early stage of fruit development than in later stages, and no energy is consumed in the synthesis or conversion of sucrose (Koch, 2004). Compared with CK, SuSy in water deficit treatment (T_1_, T_2_, and T_3_) increased by 5.51, 37.12, and 17.88%, respectively, indicating that water deficit resulted in increased SuSy activity. It was found that AI activity was greater for K_1_ than for K_0_ (Figures 4G,H), correspondingly, the hexose was also greater for K_1_ than K_0_ in the fruit (Figures 4A,B), which were consistent with the results of previous studies (Almeselmani et al., 2009; Cui et al., 2011; Cao et al., 2011).

Distribution of photosynthate in sink between sucrose and starch is directly affected by SPS activity, the lower the SPS, the less sucrose accumulation (Hubbard et al., 1991). The SPS activity showed a gradual downward trend with fruit development, but increased slightly at 73DAA, on account of the AI activity decreased at this time (Figures 4E,F), resulting in sucrose accumulation in the experiment. Especially in K_1_ treatments, sucrose increased before harvest due to the enhanced SPS activity by potassium (Figure 4D).

Interestingly, SPS behaves differently in leaves and fruits under water deficit conditions. When plants are subjected to water stress, leaves stomata are closed and osmoregulation is enhanced (Dai et al., 2007), large amounts of monosaccharides, soluble proteins, proline accumulate, and the osmotic potential is significantly reduced (Mirás-Avalos et al., 2013), which helps maintain high cell turgor (Kobashi et al., 2000) and prevents cell dehydration, which is an internal mechanism for plants to resist water stress (Srinivasa et al., 2001). Glucose and fructose, as the most important osmotic adjustment substances, were significantly increased (Lu et al., 2009). SPS activity was reduced and catabolic enzyme activity (AI) was increased due to substrate feedback inhibition, which was in line with the findings of Wu et al. (2007) on tomato leaves.

Studies have shown that tomato fruit with 8 days interval irrigation treatment had higher sucrose content than those with 6 days interval treatment, and water deficit increased SuSy and SPS activities (Han et al., 2012). Zhang et al. (2009) showed that with the intensification of water stress, the activities of SPS and SuSy showed an increasing trend in wolfberry fruit, the more significant the degree of water stress, the more favorable the sucrose accumulation. Liu et al. (2012) found that the activities of AI, SuSy, and SPS were greater for water stress than full irrigation at the late stages of litchi fruit development, which favored sugar accumulation. The results of our experiment were the same as previous studies (Figures 4C,I).

Starch maintains sink strength, ensures carbohydrate is imported from source to sink, and sustains normal development in fruit (Mohapatra et al., 2009; Jonik et al., 2012). The enzymes principally active in starch metabolism are SP and AGPase (Schaffer and Petreikov, 1997). Our results showed that in both water deficit and applying potassium treatments, SP activity was noticeably greater than in CK and that potassium enhanced starch hydrolase activity (Figures 5E,F), resulting in an increase in glucose in the fruit (Figures 5A,B), which was the same as the study of Guo et al. (2019). There was no significant difference in AGPase activity in water treatments (Table 4), but previous studies showed that enzyme activity was greater in water deficit treatments than in full irrigation (Su et al., 2015), which may be related to the later sampling time (34–73 daa). In addition, compared with K_0_ treatments, the AGPase activity in K_1_ was reduced by 10.87%, indicating that potassium application can significantly reduce the AGPase activity in the fruit, thereby inhibiting the starch synthesis and accumulation (Vardy et al., 2002).

The rate of carbon conversion indicates carbon allocation and corresponds to metabolic activity during fruit development (Alonso et al., 2007); carbon allocation is a direct measure of sugar content in the source and the sink (Gomathi and Thandapani, 2004; Dumschott et al., 2017). Sugar conversion and sugar content depend on enzyme activity and are related to the rate of carbon conversion. Our results show that greater AI activity increased sucrose hydrolysis and decreased *p*_2_(*t*) (Figure 6B), thus maintaining the sucrose concentration gradient between sink and source to ensure sugar accumulation in the fruit (Lalonde et al., 2003). The structure of the fruit stabilized as the fruit developed, and metabolic activity in the synthesis of starch [*p*_5_*_*m*_*(*t*)] and other compounds [*p*_3_(*t*)] decreased (Figure 6). AI was significantly negatively correlated with *p*_2_(*t*), *p*_3_(*t*), and *p*_5_*_*m*_*(*t*) (Figure 7).

SuSy converts sucrose into fructose and uridine diphosphate glucose (UDPG) in a reversible reaction. In the early stage, when AI activity was low, SuSy was most active in sucrose conversion (Winter and Huber, 2000). Decreased SuSy activity resulted in sucrose synthesis being blocked and a decrease in *p*_2_(*t*) (Figure 6B). SPS maintained a low level of activity throughout all growth stages and therefore has a low correlation with the rate of carbon conversion (Figure 7).

AGPase is a rate-limiting enzyme in starch synthesis (Sweetlove et al., 1999). Decrease in AGPase activity inevitably leads to a decrease in the rate of starch synthesis. Decreased AGPase activity reduces the synthesis of UDPG from glucose-1-phosphate and uridine triphosphate (Janhendrik et al., 2000). UDGP is a precursor of sucrose synthesis: UDPG and fructose-6-phosphoric acid are converted to sucrose, catalyzed by SPS (Black et al., 2006; Xu et al., 2017). Insufficient substrate eventually decreases the rate of sucrose synthesis (Figures 6B,D). Thus AGPase was positively correlated with *p*_2_(*t*) and *p*_5_*_*m*_*(*t*) (Figure 7).

Canonical correlational analysis showed that SuSy and AGPase are critical enzymes and that carbon conversion was influenced mainly by *p*_1_(*t*) in sucrose metabolism and by *p*_5_*_*m*_*(*t*) in starch metabolism. There is a one-to-one correspondence between the two enzymes in maintaining carbon balance in tomato fruit (Figure 1). SuSy regulates *p*_1_(*t*), and AGPase regulates *p*_5_*_*m*_*(*t*), as shown by canonical correlational analysis.

## Conclusion

This study shows that water stress and potassium could increase carbon supply flux in the fruit, which is beneficial to carbon accumulation. The addition of potassium modified the balance between enzymes active in sugar and starch synthesis, further reducing AGPase activity, resulted in the increase of hexose. Canonical correlational analysis showed that the carbon conversion rate was principally affected by *p*_1_(*t*) and *p*_5_*_*m*_*(*t*). Sucrose synthase and AGPase had the greatest effects on enzyme activity in tomato fruit, which showed they were critical to regulating the rate of carbon conversion. Spearman correlation analysis showed that acid invertase was significantly negatively correlated with *p*_2_(*t*) and *p*_5_*_*m*_*(*t*). Sucrose synthase was noticeably positively correlated with *p*_2_(*t*), *p*_3_(*t*), and *p*_5_*_*m*_*(*t*). AGPase was significantly positively correlated with *p*_2_(*t*) and *p*_5_*_*m*_*(*t*).

## Data Availability Statement

The raw data supporting the conclusions of this article will be made available by the authors, without undue reservation.

## Author Contributions

AL conducted the experiment and finished the first manuscript. CZ supervised the manuscript. JC assisted in the experiment. All authors contributed to the article and approved the submitted version.

## Conflict of Interest

The authors declare that the research was conducted in the absence of any commercial or financial relationships that could be construed as a potential conflict of interest.

## Supplementary Material

Supplementary Figure 1 Details of the experiment site and plant layout in the greenhouse. The greenhouse used in the experiment was a non-heated naturally ventilated solar greenhouse oriented lengthwise east–west (length 76 m, width 8 m), constructed with a PVC film canopy (0.2 mm thick) supported by a steel and bamboo strip framework. Tomato plants were spaced 60 cm between rows and 80 cm between plants within a row.
