# Supplementary material for: The Associated With Carbon Conversion Rate and Source–Sink Enzyme Activity in Tomato Fruit Subjected to Water Stress and Potassium Application
Source: Front Plant Sci. 2021 Jun 16;12:681145. doi: 10.3389/fpls.2021.681145 (PMC8245005; doi:10.3389/fpls.2021.681145)

SUPPLEMENTARY FIGURE 1 Details of the experiment site and plant layout in the greenhouse. The greenhouse used in the experiment was a non-heated naturally ventilated solar greenhouse oriented lengthwise east–west (length 76 m, width 8 m), constructed with a PVC film canopy (0.2 mm thick) supported by a steel and bamboo strip framework. Tomato plants were spaced 60 cm between rows and 80 cm between plants within a row.

SUPPLEMENTARY FIGURE 1

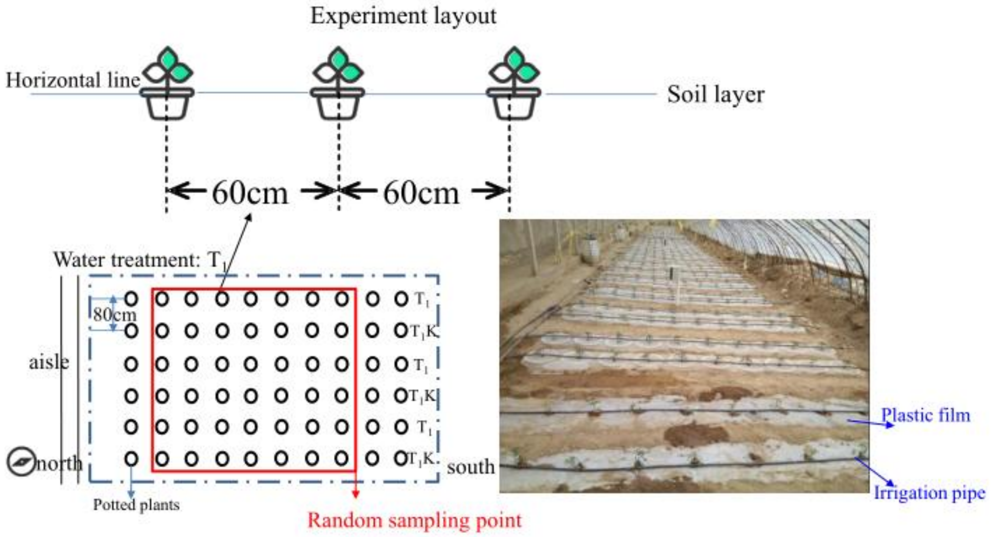

Supplement: Supplementary Figure 1 — Details of the experiment site and plant layout in the greenhouse. The greenhouse used in the experiment was a non-heated naturally ventilated solar greenhouse oriented lengthwise east–west (length 76 m, width 8 m), constructed with a PVC film canopy (0.2 mm thick) supported by a steel and bamboo strip framework. Tomato plants were spaced 60 cm between rows and 80 cm between plants within a row. [file Presentation_1.pdf]
